# PD-L1 maintains neutrophil extracellular traps release by inhibiting neutrophil autophagy in endotoxin-induced lung injury

**DOI:** 10.3389/fimmu.2022.949217

**Published:** 2022-08-09

**Authors:** Cheng-long Zhu, Jian Xie, Zhen-zhen Zhao, Peng Li, Qiang Liu, Yu Guo, Yan Meng, Xiao-jian Wan, Jin-jun Bian, Xiao-ming Deng, Jia-feng Wang

**Affiliations:** Faculty of Anesthesiology, Changhai Hospital, The Naval Medical University, Shanghai, China

**Keywords:** ARDS, PD-L1, autophagy, neutrophils, neutrophil extracellular traps, anti-PD-L1 therapy

## Abstract

Programmed death ligand 1 (PD-L1) is not only an important molecule in mediating tumor immune escape, but also regulates inflammation development. Here we showed that PD-L1 was upregulated on neutrophils in lipopolysaccharide (LPS)-induced acute respiratory distress syndrome (ARDS). Neutrophil specific knockout of PD-L1 reduced lung injury in ARDS model induced by intratracheal LPS injection. The level of NET release was reduced and autophagy is elevated by PD-L1 knockout in ARDS neutrophils both *in vivo* and *in vitro*. Inhibition of autophagy could reverse the inhibitory effect of PD-L1 knockout on NET release. PD-L1 interacted with p85 subunit of PI3K at the endoplasmic reticulum (ER) in neutrophils from ARDS patients, activating the PI3K/Akt/mTOR pathway. An extrinsic neutralizing antibody against PD-L1 showed a protective effect against ARDS. Together, PD-L1 maintains the release of NETs by regulating autophagy through the PI3K/Akt/mTOR pathway in ARDS. Anti-PD-L1 therapy may be a promising measure in treating ARDS.

## Highlights

•Upregulated PD-L1 on neutrophils contributes to NET release and acute lung injury *via* regulating autophagy through PI3K/Akt/mTOR pathway

•Anti-PD-L1 antibody administration may be a promising therapeutic strategy for ARDS

## Introduction

Acute respiratory distress syndrome (ARDS) represents respiratory dysfunction in critically ill patients. It is defined as an acute onset of non-cardiogenic pulmonary edema and hypoxemia caused by alveolar inflammation or infection that requires mechanical ventilation (1–3). The Berlin definition of ARDS classified ARDS into three categories, based on the degree of hypoxemia: mild (200 mmHg < PaO_2_/FiO_2_ ≤ 300 mmHg), moderate (100 mmHg < PaO_2_/FiO_2_ ≤ 200 mmHg), and severe (PaO_2_/FiO_2_ ≤ 100 mmHg) (2, 4). Estimates of the incidence of ARDS in high- and middle-income countries vary from 10.1 to 86.2 per 100,000 person-years in the hospital inpatients (2, 5), however, the incidence is even higher in the patients admitted to the ICU. Mortality in ARDS is high (30%-40% in most studies) (1). The challenge of treating ARDS to reduce mortality and achieving better outcomes is enormous.

Dysregulated inflammation, inappropriate accumulation and activity of leukocytes and platelets, uncontrolled activation of coagulation pathways, altered permeability of alveolar endothelial and epithelial barriers remain central pathophysiologic mechanisms in ARDS (2, 6). Neutrophils are considered to be complex cells with important functions as effectors of the innate immune response and they are able to regulate various processes such as acute injury and repair, autoimmune and chronic inflammatory processes. In addition, neutrophils can stimulate adaptive immunity, as they have been shown to activate splenic B lymphocytes (7, 8). During periods of inflammation, the rate of neutrophil production increases by 10-fold to 10^12^ cells per day, which are recruited to the site of infection, killed by phagocytosis, and then cleared by macrophages (8, 9). During ARDS, a large number of neutrophils accumulate in the lungs. The neutrophil activation, infiltration and delayed clearance are thought to play a critical role in the pathogenesis of ARDS, while the presence of excessive neutrophil extracellular traps (NETs) can lead to more severe lung injury (10–13). NETs are large, extracellular, reticular structures composed of cellular-free DNA, histones, and globular proteins such as myeloperoxidase (MPO) (14, 15), which have an important role in the clearance of systemic microbial infections. Unfortunately, the contribution of NETs in tissue damage has also been well documented in infectious diseases (15). NETs can directly kill epithelial and endothelial cells through free circulating histones and cytotoxic MPO (16, 17), which has important implications for the pathogenesis of ARDS.

Autophagy, an evolutionarily conserved cellular mechanism, degrades proteins and organelles in the lysosome and is a principal self-protection mechanism. Upon activation, multiple stimuli interfere with strictly regulated processes under a variety of pathological conditions, including hypoxia, nutrient or energy deficiencies, and even cell differentiation signals (18–20). Since autophagy is the key to cellular resistance to external stress, enhanced autophagy can protect against sepsis-induced dysfunction of kidney (21), lung (22, 23), liver (24) and other organs. Interestingly, recent evidence confirmed that LC3 overexpression attenuated acute lung injury in septic mice (25). In addition, the protective effect of glyceraldehyde-3-phosphate dehydrogenase (GAPDH) against sepsis-associated lung injury is mediated by enhanced ATG12-dependent autophagy (26, 27). Recent studies have implicated autophagy as a key contributor to the membrane changes observed during NETosis (28). However, the autophagy status in neutrophils of ARDS patients, and its association with NETosis, are largely unknown.

Programmed death ligand 1 (PD-L1), which is upregulated upon activation in bone marrow cells, lymphocytes, normal epithelial cells and cancer cells, is an important target for immune checkpoint blockade therapy (29–33). PD-L1 blockade can exert a protective effect on sepsis at least partly by inhibiting lymphocyte apoptosis and reversing monocyte dysfunction (34). We and others have previously shown that PD-L1 is upregulated in neutrophils and may be a potential biomarker for sepsis-induced immunosuppression (35). Furthermore, increased expression of PD-L1 on human neutrophils delays apoptosis, maintains phosphorylation of Akt and drives sepsis-induced lung injury (36). The PI3K/Akt pathway has an important role in maintaining neutrophil survival by PD-L1. It has been implicated in the regulation of lung injury, and inhibition of this pathway may reduce lung injury (35, 37). However, the protective effect of PD-L1 deficiency in neutrophils against sepsis-induced lung injury may be also attributed to reversal of sepsis-induced immunosuppression and enhanced clearance of bacteria. Therefore, the present study was performed to investigate the direct role of PD-L1 in neutrophil autophagy, NET release and LPS-induced lung injury. The potential role of PD-L1 as a therapeutic target against acute lung injury was also investigated using a PD-L1-neutralizing antibody.

## Materials and methods

### Mice

Neutrophil specific PD-L1 conditional knockout (CKO) mice were generated by crossing PD-L1^flox/flox^, engineered using CRISPR/Cas9 (BIORAY LABORATORIES Inc., Shanghai, China), with elane (Ela)^cre/cre^ mice purchased from the EMMA mouse repository (INFRAFRONTIER, München, Germany) (36, 38, 39). Male C57BL/6J mice that were 6-8 weeks of age and free of specific pathogens were obtained from the Research Animal Center of Navy Medical University (Shanghai, China). The mice were housed in barrier cages under controlled environmental conditions (12/12-h light/dark cycle; 55% ± 5% humidity; 23°C). All animal studies were approved by the Committee on Ethics of Biomedicine Research in Naval Medical University, Shanghai, China.

### Patients

Patients fulfilling the clinical criteria for ARDS were recruited from the Central Intensive Care Unit of Changhai Hospital, Shanghai, China. Healthy donors served as controls. Peripheral blood samples were collected from ARDS patients within the first 24 h of admission. The study protocol was approved by the Committee on Ethics of Biomedicine Research in Naval Medical University, Shanghai, China.

### *In vivo* experiments

The ARDS model was established as described previously (40). Briefly, the mice were anesthetized with sevoflurane (Hengrui, Lianyungang, Jiangsu, China). After exposing the trachea, a trimmed sterile 31-gauge needle was inserted into the tracheal lumen. LPS (Sigma, St Louis, MO, USA) diluted in endotoxin-free saline was intratracheally (IT) injected at a dose of 10 mg/kg in 100 μl saline. To treat ARDS mice, anti-PD-L1 antibody (eBiosciences, San Diego, CA, USA) was intraperitoneally administered at a dose of 50 μg/mouse immediately after the injection of LPS. Hematoxylin-eosin staining was conducted to quantify lung injury and the result was semi-quantified by two independent pathologists according to the criteria reported previously (41, 42). The cells in the BALF (BALF was obtained by intratracheal injection with 1 ml cold PBS) were collected and stained with anti-Ly6G-PE and anti-CD11b-APC (eBiosciences, San Diego, CA, USA) to detect neutrophils by flow cytometry. BALF levels of TNF-α, IL-1β and IL-6 were detected by enzyme linked immunosorbent assay (ELISA, R&D, Minneapolis, MN, USA). The wet-to-dry weight (W/D) ratio was calculated to assess the edema. The protein concentration in the BALF was assessed with a BCA detection kit (Thermo Scientific, Rockford, IL, USA).

### Neutrophil purification, stimulation and transfection

The mice neutrophils were isolated from bone marrow by positive selection magnetic cell separation (MACS) using the Miltenyi Biotec mouse AntiLy-6G MicroBead Kit according to the manufacturer’s instructions (Miltenyi, Bergisch Gladbach, Germany) (43). The purity of the isolated cells was at least 95% according to the expression of Gr-1 and CD11b. Human neutrophils were purified by density gradient centrifugation with 3% Dextran and Ficoll-Hypaque (GE Healthcare, Little Chalfont, UK) as previously described (44, 45). The cells were resuspended in DMEM supplemented with 10% FBS, 1% glutamine, and 1% penicillin/streptomycin solution at a concentration of 1 x 10^6^ cells/mL. Cells were incubated in polypropylene tubes to prevent adherence. The purity of the isolated cells was at least 96% according to the expression of CD15. Neutrophils were stimulated with LPS (1ug/ml) (Sigma, St Louis, MO, USA) and IFN-γ (10ng/ml)(Peprotech, Rocky Hill, USA) for 21hours. PD-L1 siRNA transfection was performed using the Santa Cruz siRNA transfection reagent for 21 hours according to the manufacturer’s instructions (Dallas, TX, USA).

### MPO-DNA (NETs) assay

To quantify NETs in mouse BALF and in cell culture supernatant, a capture ELISA based on MPO associated with DNA was applied (46). For the capture antibody, 5 μg/ml anti-MPO Ab (Invitrogen, Carlsbad, CA, USA) was coated onto 96-well plates (dilution 1: 500 in 50 μl) overnight at 4°C. After washing 3 times (300 μl each), 20 μl of samples was added to the wells with 80 μl incubation buffer containing a peroxidase-labeled anti-DNA antibody (Cell Death ELISA PLUS, Roche, Indianapolis, IN, USA; dilution 1: 25). The plate was incubated for 2 hours, shaken at 300 rpm at room temperature. After 3 washes (300 μl each), 100 μl peroxidase substrate (ABTS) was added. Absorbance at 405 nm wavelength was measured after 20 minutes of incubation at room temperature in the dark. Results are reported as percent of WT mice BALF or healthy cell culture supernatant ± SD, arbitrarily set at 100%.

### Flow cytometry

Mice were euthanized 24 h after LPS ARDS or sham-operated surgery to get BALF. Cells were stained with fluorochrome-conjugated anti-Gr-1, anti-PD-L1 antibodies (eBioscience San Jose, CA, USA). Flow cytometric analysis was performed on a MACS Quant (Miltenyi Biotech, Bergisch Gladbach, Germany) using Flowjo software version 7.6 (Tree Star, Ashland, OR, USA).

### Western blot and immunoprecipitation

Western blotting was performed to detect PD-L1, LC3B, Beclin-1, p110 and p85, Akt, mTOR phosphorylation. The antibodies included anti-PD-L1 (Santa Cruz, Dallas, TX, USA), anti-LC3B (CST, Princeton, NJ, USA), anti-Beclin-1 (CST, Princeton, NJ, USA), anti-p85 (Abcam, Cambridge, MA, USA), anti-phosphorylated p85 (Y607) (Abcam, Cambridge, MA, USA), anti-Akt (CST, Princeton, NJ, USA), anti-phosphorylated Akt (S473) (CST, Princeton, NJ, USA), anti-mTOR (CST, Princeton, NJ, USA), anti-phosphorylated mTOR (CST, Princeton, NJ, USA) and anti-β-actin (CST). Immunoprecipitation assay was performed using the anti-p110 antibodies (CST, Princeton, NJ, USA) and Protein7 G beads (GE Healthcare, Mississauga, ON, Canada). LC3B II/I intensity was calculated using Image J software.

### Immunofluorescence

Following anaesthetized, animals were perfused transcardially by phosphate-buffer saline (PBS) and 4℅ paraformaldehyde (PFA) successively. Lungs were removed and fixed in PFA at 4°C for more than 24 h. Thereafter, lungs were immersed in 30% sucrose for dehydration and then cut into 30-μm-thick slices on a cryostat. Tissue sections were incubated with 0.5℅ Triton X-100 for 20 min for permeation, and subsequently immersed in 3℅ bovine serum albumin for 1 h at room temperature for blocking. The sections were then incubated with primary antibodies (1:200) overnight at 4°C. After washed with PBS three times, the sections were incubated with secondary antibodies (1:1000) for 1 h at room temperature. Following activation of neutrophils, the cells were collected, washed with PBS for three times, fixed with 4% paraformaldehyde for 20 min, permeabilized with 0.1% Triton X-100 for 30 min at room temperature, and then blocked with 1% bovine serum albumin (BSA) in distilled water for 30 min. The cells were incubated overnight with a mouse anti- MPO antibody (Abcam cat. Ab25989) at a 1:500 dilution, an anti-Histone 3 antibody (Abcam; cat. Ab5103) at a 1:500 dilution. After being washed with PBS three times, the cells were stained with secondary antibodies, including Alexa Fluor 488-conjugated anti-rabbit and Alexa Fluor 594-conjugated anti-mouse antibodies. We used DAPI as a nuclear counterstain (Life Technologies). After being rinsed and mounted with glycerol, the sections were recorded using fluorescence microscopy.

### Electron microscopy

Autophagosomes were observed in neutrophils in mice BALF by transmission electron microscopy. NETs were observed in human neutrophils by scanning electron microscopy. Cells were fixed with 2.5% glutaraldehyde, postfixed with 1% osmiumtetroxide, contrasted with uranylacetate and tannic acid, dehydrated, and embedded in Polybed (Polysciences).

### Fluorescence microscopy

Neutrophils LC3B puncta were assessed with fluorescence Confocal to evaluate autophagy. Confocal fluorescence images were captured using a Leica TCS SPE confocal fluorescence microscope (Leica Microsystems). Non-confocal images were acquired with an Axio Observer inverted fluorescence microscope (Carl Zeiss). LC3B puncta was calculated using Image J software.

### Statistical analysis

All statistical analyses were perform using Graphpad Prism 8.0 (San Diego, CA, USA). Comparisons of normally distributed continuous data were performed with student’s t test or one-way analysis of variance. The lung injury score was compared using the Kruskal-Wallis test with Dunn’s adjustment for multiple comparisons. A p <0.05 was considered as statistically significant.

## Results

### Upregulated PD-L1 promotes neutrophil recruitment in lung and aggravates lung injury

**Figure 1:**
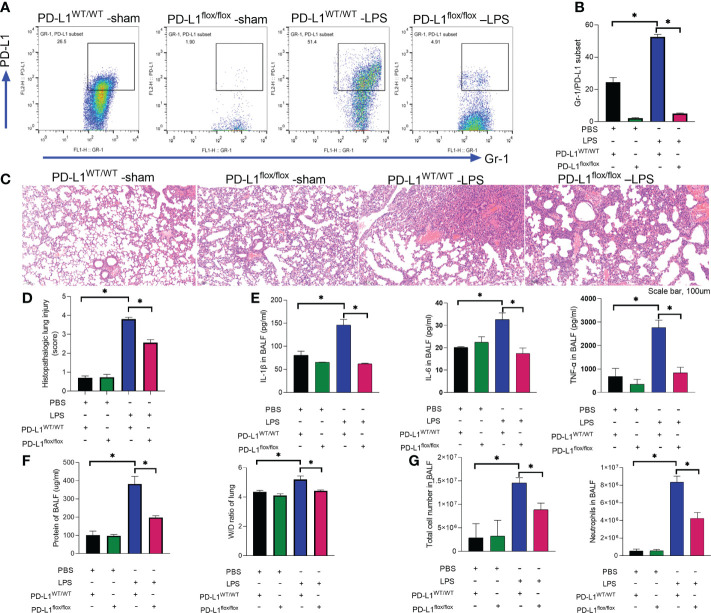
Up-regulated PD-L1 has a facilitating effect on the pathogenesis of ARDS and that a lack of PD-L1 has a protective effect. **(A–G)** PD-L1WT/WT mice and PD-L1flox/flox mice were injected intratracheally with LPS (10mg/kg) for 24h. **(A, B)** Percent of PD-L1 expression on neutrophils in BALF. **(C)** Representative histological section of the lungs was stained by HE staining (scale bar:100um). **(D)** The lung injury scores were determined. **(E)** TNF-α, IL-1β, IL-6 levels in BALF. **(F)** BCA assay was used to determine the total protein concentration in BALF and lung tissues were weighed to calculate the W/D ratio. **(G)** The total cells and neutrophils detected by flow cytometry in BALF. The values presented are mean ± SEM (n=6; *P<0.05, one-way analysis of variance).

We previously reported that PD-L1 expression was increased in neutrophils during sepsis, and PD-L1 could inhibit the apoptosis of neutrophils through the PI3K/Akt pathway (36), leading to increased neutrophils aggregation in the lungs to aggravate lung injury during sepsis. But the *in vivo* role of neutrophil PD-L1 against ARDS might be confused by its role in immunosuppression. To investigate whether PD-L1 plays a direct role in acute lung injury, we introduced the neutrophil-specific PD-L1 knockout (PD-L1^flox/flox^) mice and PD-L1^WT/WT^ mice to establish an ARDS model by intratracheal LPS injection (47). After LPS stimulation, the expression of PD-L1 in lung neutrophils increased, but compared with PD-L1^WT/WT^ mice, the increase of PD-L1 in lung neutrophils of PD-L1^flox/flox^ mice was not significant (**Figures 1A, B**). LPS-induced lung injury was present in both PD-L1^flox/flox^ and PD-L1^WT/WT^ mice, characterized as alveolar wall thickening, interstitial and alveolar infiltration of inflammatory cells, and hemorrhage in the lungs. However, LPS injection caused more severe lung injury in PD-L1^WT/WT^ mice compared with the PD-L1^flox/flox^ mice (**Figures 1C, D**). Inflammatory cytokines are usually elevated in the lungs in LPS-induced ARDS. Compared with the PD-L1^WT/WT^ mice, LPS significantly attenuated the secretion of TNF-*α*, IL-1β and IL-6 in BALF of PD-L1^flox/flox^ mice (**Figure 1E**). In ARDS, there is increased infiltration of macromolecules and fluids into the interstitium due to a compromised endothelial cell barrier (3). We measured the wet-to-dry weight ratio of the lungs and total protein concentration of BALF which is a signal of pulmonary edema in ARDS. Neutrophil-specific PD-L1 knockout effectively attenuated the effect of LPS in increasing wet-to-dry ratio of lung and increase total protein concentration of BALF (**Figure 1F**). Secretion of pro-inflammatory cytokines by resident alveolar macrophages in ARDS leads to recruitment of neutrophils and monocytes or macrophages, activation of alveolar epithelial cells and effector T cells, and promoting inflammation and tissue injury (2). It was observed that LPS increased the number of total cells and neutrophils in BALF from PD-L1^WT/WT^ mice. Similarly, this trend was attenuated in the PD-L1^flox/flox^ mice (**Figure 1G**). Our data suggested that up-regulated PD-L1 has a facilitating effect on the pathogenesis of ARDS and that the lack of PD-L1 may reduce neutrophil recruitment and inflammatory responses in the lung.

### PD-L1 modulates the release of NETs both *in vivo* and *in vitro*

**Figure 2:**
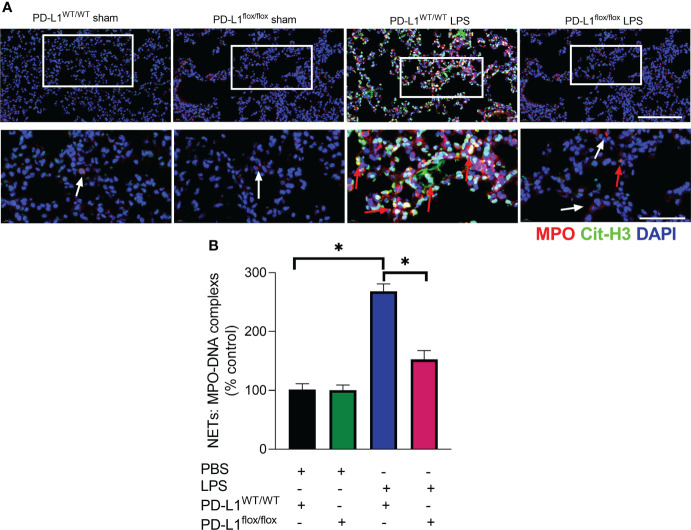
Genetic deletion of PD-L1 in neutrophils can reduce NET release in ARDS model. **(A, B)** PD-L1WT/WT mice and PD-L1flox/flox mice were injected intratracheally with LPS (10mg/kg) for 24h. **(A)** Representative immunofluorescence images of Cit-H3 (green) and MPO (red) staining with blue DAPI nuclear staining in lungs. Neutrophils express MPO (red) and NET forming neutrophils also express Cit-H3 (green). Cyan fluorescence represents the colocalization of Cit-H3 with DNA. The white arrowheads point to neutrophils not making NETs and the red arrows to neutrophils making NETs. The scale bar indicates 20 μm. Higher magnification images are shown lower row of figures – scale bars indicate 10 μm. **(B)** MPO-DNA complex measured in NETs structures in BALF. The values presented are mean ± SEM (n=3; *P<0.05, one-way analysis of variance).

**Figure 3:**
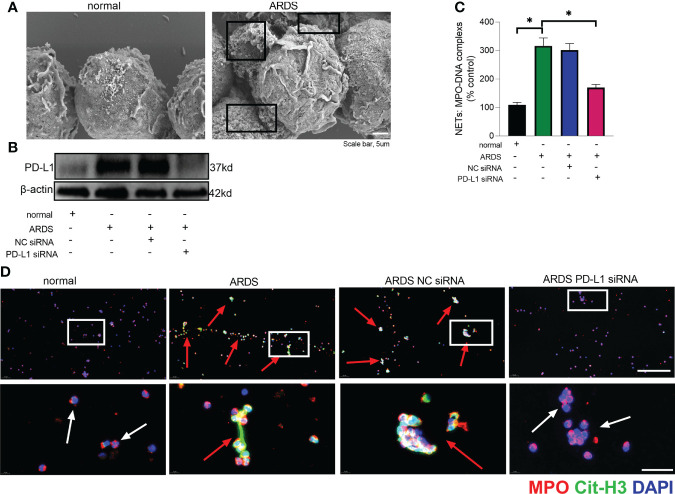
Down-regulated PD-L1 of neutrophils reduces NET release in human. **(A)** Representative scanning electron micrographs of neutrophil NET of normal and ARDS patients (scale bar: 5um). **(B)** Genetic silencing of PD-L1 using siRNA in ARDS neutrophils for 21 hours significantly decreased PD-L1 expression. **(C)** MPO-DNA complex measured in NETs structures in neutrophils culture supernatant. **(D)** Representative immunofluorescence images of Cit-H3 (green) and MPO (red) staining with blue DAPI nuclear staining in neutrophils. Neutrophils express MPO (red) and NET forming neutrophils also express Cit-H3 (green). Cyan fluorescence represents the colocalization of Cit-H3 with DNA. The white arrowheads point to neutrophils not making NETs and the red arrows to neutrophils making NETs. The scale bar indicates 50 μm. Higher magnification images are shown lower row of figures – scale bars indicate 10 μm. The values presented are mean ± SEM (n=6; *P<0.05, one-way analysis of variance).

The prerequisite for NETs formation is the activation of neutrophils and the release of their DNA. Excessive NETs in inflammation can be injurious to tissues, and targeted modulation of NET release is a new direction in the treatment of acute lung injury (48–50). The *in vivo* data showed that increased release of NETs in the lungs of LPS-challenged mice and excessive release of NETs was reversed in the lung when PD-L1 was knocked out specifically in neutrophils (**Figures 2A, B**). In addition, we stimulated bone marrow neutrophils of mice with LPS/IFN-γ and found that the knockout of PD-L1 resulted in a reduction in neutrophil production of NETs *in vitro* (**Supplementary Figure 1**), indicating that down-regulated PD-L1 can prevent overproduction of NETs. We also collected peripheral blood neutrophils from ARDS patients and silenced PD-L1 expression using siRNA to detect PD-L1 expression and NET release (**Figures 3A–D**). The results showed that neutrophil NET release was increased in ARDS patients compared to healthy volunteers, while NET release was reduced in PD-L1-knockdown neutrophils.

### PD-L1 modulates the release of NETs by regulating autophagy

**Figure 4:**
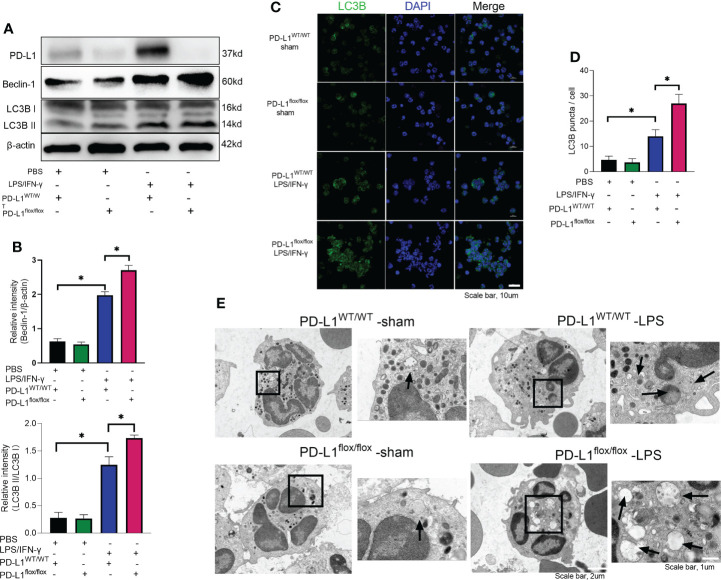
Genetic deletion of PD-L1 can increase autophagy levels. **(A–D)** Neutrophils from PD-L1WT/WT mice or PD-L1flox/flox mice are stimulated with IFN-γ (10ng/ml) and LPS (1μg/ml) for 21 hours. **(A)** PD-L1, Beclin-1 and LC3B II/I immunoblotting in neutrophils. **(B)** Integrated optical density ratio of LC3B II/LC3B (I) **(C)** Autophagy induction assessed with LC3B staining (confocal microscopy; green: LC3B; blue: DNA) in neutrophils (scale bar: 10um). **(D)** LC3B puncta/cell are depicted. **(E)** PD-L1WT/WT mice and PD-L1flox/flox mice were injected intratracheally with LPS (10mg/kg) for 24h. Representative transmission electron micrographs of neutrophil autophagosomes in BALF (scale bar: 2um, 1um). The values presented are mean ± SEM (n=6; *P<0.05, one-way analysis of variance).

In chronic kidney disease, excessive NETs are closely associated with endothelial cell dysfunction. Levels of NETs were significantly increased after autophagy was inhibited, suggesting a protective role of autophagy in excessive NET formation (51). In the present work, we observed that LPS/IFN-γ stimulation significantly augmented the level of autophagy in neutrophils, but PD-L1-knockdown neutrophils have a higher level of autophagy (**Figures 4A, B**). And we were able to confirm more accumulation of localized LC3B puncta in PD-L1-knockdown neutrophils by confocal microscopy (**Figures 4C, D**), indicating that autophagy levels are further enhanced after knockdown of PD-L1 in neutrophils. To verify whether neutrophil PD-L1 regulated autophagy in ARDS, we confirmed that the neutrophil autophagosomes in BALF of ARDS PD-L1^flox/flox^ mice were more than those of PD-L1^WT/WT^ mice by electron microscopy, which implied that PD-L1-knockdown neutrophils had a higher level of autophagy (**Figure 4E**). These results may suggest that PD-L1 inhibits autophagy potential in neutrophils and PD-L1 knockdown can lift this inhibition to produce more autophagy under ARDS.

Next step, autophagy was inhibited by wortmannin administration (52). Interestingly, when autophagy was inhibited by wortmannin, the release of NETs was re-introduced in lungs of PD-L1^flox/flox^ mice treated with LPS (**Supplementary Figure 2**). Similar trend was also observed in neutrophils *in vitro*. The inhibition of NET release by PD-L1 knockout was reversed in mice neutrophils by wortmannin stimulated with LPS and IFN-γ (**Supplementary Figure 3**).

### PD-L1 regulates autophagy through PI3K/Akt/mTOR pathway

**Figure 5:**
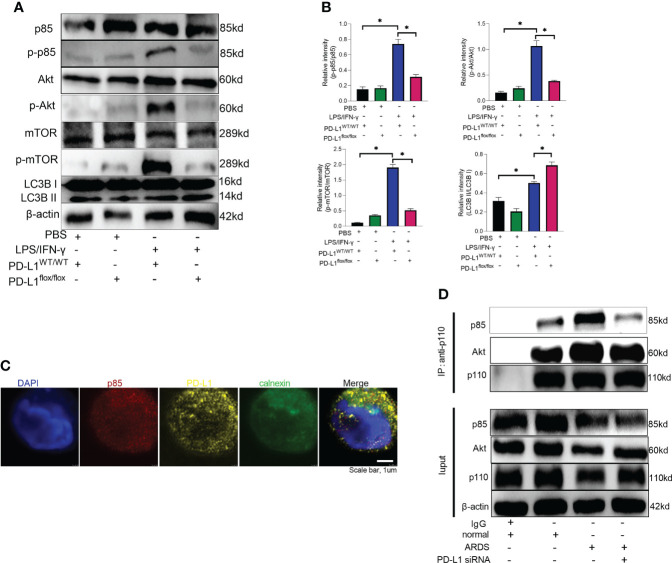
PD-L1 binds to p85 on the ER to regulate autophagy *via* PI3K/Akt/mTOR pathway. **(A)** Neutrophils from PD-L1WT/WT mice or PD-L1flox/flox mice are stimulated with IFN-γ (10ng/ml) and LPS (1μg/ml) for 21 hours. Protein levels of p85, p-p85, Akt, p-Akt, mTOR, p-mTOR and LC3B II/I in neutrophils were evaluated by western blot analysis. **(B)** Integrated optical density ratio of p-p85/p85, p-Akt/Akt, P-mTOR/mTOR, and LC3B II/LC3B (I) The values presented are mean ± SEM (n=3; *P<0.05, one-way analysis of variance). **(C)** PD-L1 and p85 overlap on the ER in neutrophils from ARDS patients, which was confirmed by confocal microscopy (blue: DNA; red: p85; yellow: PD-L1; green: calnexin (ER marker)) (scale bar: 1um). **(D)** Neutrophils were transfected with PD-L1 siRNA. Immunoprecipitating p110 demonstrates that p110 complexes with more p85 and Akt in ARDS patients. The values presented are mean ± SEM (n=3; *P<0.05, one-way analysis of variance).

Mammalian target of rapamycin (mTOR), a serine/threonine kinase, plays a critical role in regulating autophagy, and its activation can inhibit autophagy (53, 54). It has been demonstrated that PD-L1 inhibition in tumors decreases PI3K/Akt/mTOR pathway activity and enhances autophagy (55, 56), but the pathways through which PD-L1 regulates autophagy in ARDS are not yet clear. Our previous work has demonstrated that PD-L1 can bind to PI3K regulatory subunit p85 in neutrophils (36), so we speculated that PD-L1 regulates autophagy *via* PI3K/Akt/mTOR signaling pathway, thus preventing the release of excessive NETs to improve ARDS. To confirm this prediction, we investigated PI3K/Akt/mTOR pathway *in vitro* with purified bone marrow neutrophils of mice. Under LPS/IFN-γ stimulation, PD-L1 expression was elevated (**Figure 4A**), PI3K/Akt/mTOR pathway activity was augmented and autophagy was enhanced in neutrophils from control mice, but in PD-L1-knockout neutrophils from mice, PI3K/Akt/mTOR pathway activity was relatively lower and autophagy was stronger (**Figures 5A, B**).

Based on our previous work (36), we explored the site of intracellular binding of PD-L1 and PI3K regulatory subunit p85 in neutrophil. PD-L1 is a transmembrane protein and OTUB1 inhibits its degradation *via* ERAD (endoplasmic reticulum-associated degradation) pathway before it is fully matured, and only the intracellular domain of ER-associated PD-L1 exposes to the cytosol (57). PI3K is composed of the p85 regulatory subunit and the p110 catalytic subunit. The p85 subunit has both inhibitory and stabilizing effects on p110. Therefore, we explored whether PD-L1 and p85 combined at the endoplasmic reticulum in neutrophil to activate the PI3K/Akt/mTOR pathway. When activated, p85 phosphorylation decreased its inhibitory effect on p110, leading to an enhanced activity of p110. Unbound p85 has an inhibitory effect on p85/p110 heterodimer (58–61). The immunofluorescence assay demonstrated that PD-L1, p85 and the ER marker calnexin colocalized in neutrophils of ARDS patients (**Figure 5C**). In addition, we investigated the interaction between p110 and p85 or Akt by immunoprecipitation in neutrophils of ARDS patients. The results showed that both p110 and p85 or Akt binding were elevated in neutrophils stimulated by LPS/IFN-γ. When PD-L1 was knocked down by siRNA, the interaction between p110 and p85 or Akt was inhibited (**Figure 5D**). These findings suggested that elevated PD-L1 expression in neutrophils from ARDS patients activated PI3K and enhanced activation of Akt and mTOR.

**Figure 6:**
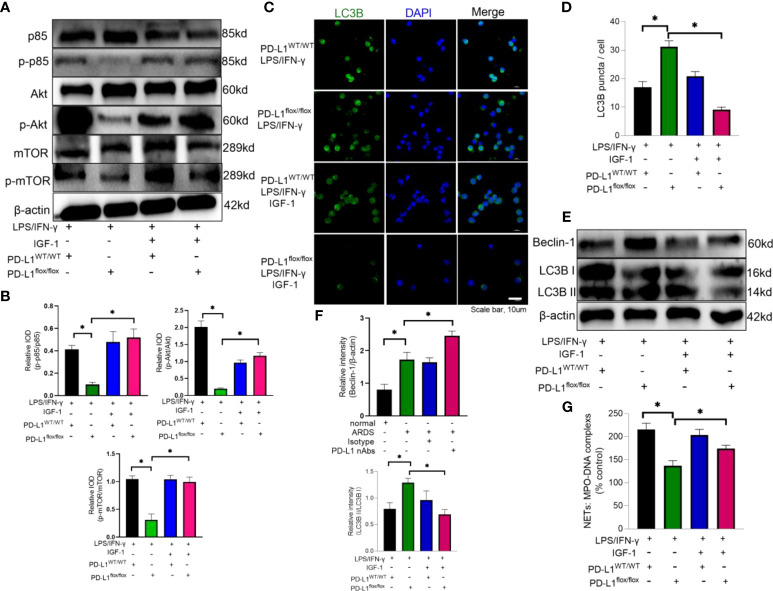
IGF-1 is able to counteract the protective effect of PD-L1 knockdown by activating the PI3K/Akt/mTOR pathway. **(A–G)** Neutrophils from PD-L1^WT/WT^ mice or PD-L1^flox/flox^ mice stimulated with IFN-g (10ng/ml) and LPS (1mg/ml) are treated with IGF-1 (10ng/ml) or DMSO for 21h. **(A)** IGF-1 can activate the PI3K/Akt/mTOR pathway confirmed by Protein levels of p85, p-p85, Akt, p-Akt, mTOR, p-mTOR in neutrophils. **(B)** Integrated optical density ratio of p-p85/p85, p-Akt/Akt, P-mTOR/mTOR. **(C)** Autophagy induction assessed with LC3B staining (confocal microscopy; green: LC3B; blue: DNA) in neutrophils (scale bar: 10um). **(D)** LC3B puncta/cell are depicted. **(E)** PD-L1, Beclin-1 and LC3B II/I immunoblotting in neutrophils. **(F)** Integrated optical density ratio of LC3B II/LC3B I. **(G)** MPO-DNA complex measured in NETs structures in neutrophils culture supernatant. The values presented are mean ± SEM (n=6; *P<0.05, one-way analysis of variance).

Insulin-like growth factor 1 (IGF-1) can activate PI3K pathway (62). Therefore, IGF-1 was introduced to reverse the effect of PD-L1 knockout on PI3K/Akt/mTOR pathway. Neutrophils from PD-L1-knockout mice were stimulated by LPS/IFN-γ and treated with IGF-1. Administration of IGF-1 increased the phosphorylation of p85, Akt and mTOR (**Figures 6A, B**), and inhibited the level of autophagy in PD-L1^flox/flox^ neutrophils (**Figures 6C–F**). And activation of PI3K/Akt/mTOR pathway also reversed the inhibition of NET release by PD-L1 knockout (**Figure 6G**). These data may imply that PD-L1 inhibits neutrophil autophagy by maintaining the activation of PI3K/Akt/mTOR pathway to curb autophagy potential under ARDS.

### Anti-PD-L1 antibody ameliorates ARDS and enhances autophagy in mice

**Figure 7:**
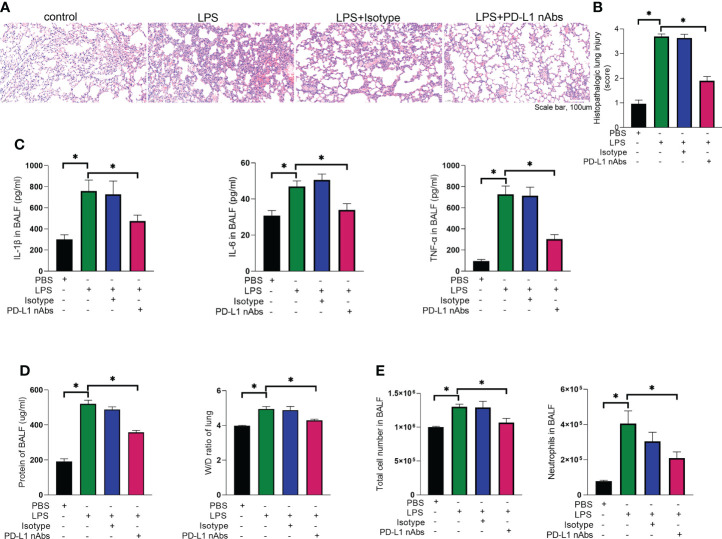
Anti-PD-L1 antibody has an ameliorating effect on the pathogenesis of ARDS. **(A–E)** C57BL/6 mice injected intratracheally with LPS (10mg/kg) were treated with anti-PD-L1 antibody (50ug/mouse) or Isotype antibody for 24h. **(A)** Representative histological section of the lungs was stained by HE staining, magnification (bar=100um). **(B)** The lung injury scores were determined. **(C)** IL-1β, IL-6, TNF-α in BALF. **(D)** BCA assay was used to determine the total protein concentration in BALF and lung tissues were weighed to calculate the W/D ratio. **(E)** The total cells and neutrophils detected by flow cytometry in BALF. The values presented are mean ± SEM (n=6; *P<0.05, one-way analysis of variance).

**Figure 8:**
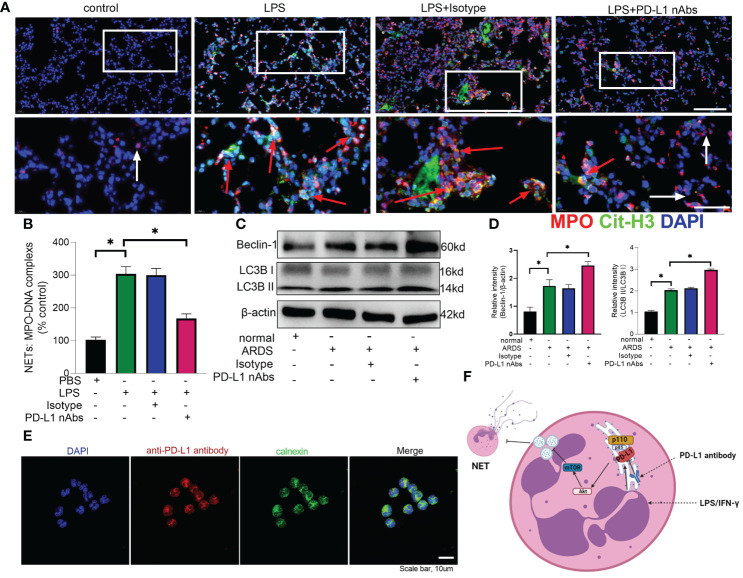
Anti-PD-L1 antibody can reduce NET release and increase autophagy levels in neutrophils *via* the PD-L1-PI3K/Akt/mTOR-autophagy-NETs pathway. **(A, B)** C57BL/6 mice injected intratracheally with LPS (10mg/kg) were treated with anti-PD-L1 antibody (50ug/mouse) or Isotype antibody for 24h. **(A)** Representative immunofluorescence images of Cit H3 (green) and MPO (red) staining with blue DAPI nuclear staining in lungs. Neutrophils express MPO (red) and NETs forming neutrophils also express Cit-H3 (green). Cyan fluorescence represents the colocalization of Cit-H3 with DNA. The white arrowheads point to neutrophils not making NETs and the red arrows to neutrophils making NETs. The scale bar indicates 20 μm. Higher magnification images are shown lower row of figures – scale bars indicate 10 μm. **(B)** MPO-DNA complex measured in NETs structures in BALF. **(C)** Neutrophils from ARDS patients were treated with anti-PD-L1 antibody (10ug/106 cells) for 21h, Beclin-1 and LC3B II/I immunoblotting in neutrophils. **(D)** Integrated optical density ratio of LC3B II/LC3B (I) **(E)** Neutrophils from ARDS patients were treated with anti-PD-L1 antibody (10ug/106 cells) for 24h, which were located on the ER (confocal microscopy; blue: DNA; red: PD-L1 antibody; green: calnexin (ER marker)) (scale bar: 10um). **(F)** When neutrophils receive LPS/IFN-γ stimulation, PD-L1 expression is elevated and binds to PI3K regulatory subunit p85 at the ER. Phosphorylated p85 binds tightly to catalytic subunit p110 to phosphorylate downstream Akt/mTOR. mTOR upregulation inhibits autophagy, which leads to the release of excessive NETs from the neutrophils. PD-L1 antibody enters the neutrophils at the ER to inhibit PD-L1 catalysis downstream of PI3K. The values presented are mean ± SEM (n=6; *P<0.05, one-way analysis of variance).

As described above, knockdown of neutrophil PD-L1 was protective against ARDS in mice through enhanced autophagy. Therefore, we investigated whether anti-PD-L1 antibody had a direct protective effect against ARDS. Anti-PD-L1 antibody was administered intraperitoneally into the murine model of ARDS and showed that lung injury, inflammatory factors, BALF protein concentration, BALF total cell count and neutrophil count were significantly lower in the anti-PD-L1 antibody group compared with those in the isotype antibody group (**Figures 7A–E**), indicating that treatment with anti-PD-L1 antibody attenuated lung injury and inhibited inflammatory factors, protein exudation and neutrophil infiltration of lung tissue. Moreover, lung NETs fluorescence and MPO-DNA ELISA assay of BALF showed that the administration of anti-PD-L1 antibody reduced the production of NETs in the lungs of ARDS mice (**Figures 8A, B**), thus effectively alleviating the damage to the lungs. In addition, we also examined beclin-1, LC3B (autophagy marker) protein with ARDS patient neutrophils, and the results indicated that autophagy was elevated after the application of anti-PD-L1 antibody (**Figures 8C ,D** and **Supplementary Figure 4**), suggesting that anti-PD-L1 antibody might exert a protective effect by increasing the level of autophagy in ARDS. Conventionally, anti-PD-L1 antibody acted on the cell surface to prevent the interaction between PD-L1 and its receptor, PD-1. We questioned how anti-PD-L1 antibody inhibited the intracellular effect of PD-L1 and the subsequent signaling molecules. Thus, we visualized the localization of anti-PD-L1 antibody in the neutrophils. We first stimulated neutrophils with a neutralizing anti-PD-L1 antibody, and then stained this neutralizing antibody. Interestingly, the immunofluorescence assay showed that anti-PD-L1 antibody was colocalized with the ER marker, calnexin (**Figure 8E**).

## Discussion

Our present data implicates that PD-L1-PI3K/Akt/mTOR-autophagy-NETs pathway plays an important role in neutrophil-mediated lung injury during ARDS (**Figure 8F**). PD-L1 expression is elevated in neutrophils from ARDS patients and mice to activate the PI3K/Akt/mTOR pathway. When PD-L1 expression is downregulated in activated neutrophils, the activation of PI3K/Akt/mTOR pathway is compromised, followed by a weakened inhibitory effect of mTOR on autophagy, which attenuates the release of excessive NETs. More importantly, our data demonstrates that PD-L1 interact with PI3K at the ER level, but not at the cell membrane as conventionally acknowledged. Furthermore, the neutralizing antibody against PD-L1 shows a direct protective effect against LPS-induced lung injury in mice.

PD-L1 is not only an important molecule in mediating tumor immune escape, but also has an important role in inflammation development. Thanabalasuriar et al. (63) reported the role of PD-L1^+^ neutrophils in mice with airway inflammation and found a protective effect of anti-PD-L1 antibody against inflammation. Our previous work presented that upregulation of neutrophil PD-L1 in inflammatory states can delay the apoptotic process through the PI3K/Akt pathway and consequently induce lung injury (35). In that study, it was observed that enhanced Akt phosphorylation in neutrophils from patients with sepsis, which was reversed by PD-L1 siRNA. It was also discovered that PD-L1 interacts with p85 subunit by immunoprecipitation (35). Herein, we demonstrated that when neutrophils were stimulated by LPS/IFN-γ, PD-L1 expression was elevated and binded to p85 subunit at the ER. Traditionally, PD-L1 is thought to be expressed on the neutrophil membranes and interacts to PD-1 on the T cells membranes, suppressing normal T cell immunity and promoting immunosuppression (35, 64, 65). In this study, we found that PD-L1 and p85 could interact with each other at the ER by colocalization under fluorescence image, thereby activating the PI3K/Akt/mTOR pathway in the host cells which expressed PD-L1. Phosphorylated p85 binds tightly to catalytic subunit p110 to phosphorylate downstream Akt/mTOR. PI3K/Akt/mTOR pathway can regulate autophagy, and mTOR inhibitors can enhance autophagy. Neutrophil PI3K/Akt/mTOR pathway activity is inhibited after knockout of neutrophil PD-L1. Pehote et al. (66) showed that enhanced autophagy might reduce immune disorder and organ deterioration in ARDS. When PD-L1 was knocked out specifically in neutrophils, autophagy was enhanced by lifting suppressed autophagy potential due to increased PD-L1 and NET release was reduced. Inhibition of autophagy by inhibitor would reverse the elevated NET release in activated neutrophils where PD-L1 expression was upregulated. This means that PD-L1 can inhibit autophagy potential (higher autophagy level by PD-L1 knockout) and maintain NET release by activating PI3K/Akt/mTOR pathway.

Autophagy and NET formation are not fully understood. Several groups have reported a requirement for autophagy in the formation of NETs (67, 68), and inhibition of autophagy prevents intracellular chromatin decondensation, which is essential for NETosis and NET formation (28). Others have reported that NETs can occur in absence of essential autophagy gene (69). Meanwhile, we discussed that NETs are released in excess during ARDS and that the increased autophagy stimulated by knockdown of PD-L1 can inhibit NET release. The role of NETs in infection control was first described in 2004 by Brinkmann et al. (70). However, over the past decade, many groups have reported on the role of NETs as a double-edged sword. In concert with their bactericidal activity, NETs also display toxic effects on host tissues in different diseases, such as diabetes, rheumatoid arthritis and sepsis (17, 71–73). In addition, at least 2 different mechanisms of NET formation have been described, including a suicide lytic NETosis and a live cell or vital NETosis (74). Since autophagy has waste disposal functions, its activation and inhibition may become a new therapeutic strategy for diseases (75). There is growing evidence that autophagy promotes cellular senescence and cell surface antigen presentation, protects against genomic instability and prevents necrosis, in addition to eliminated intracellular aggregates and damaged organelles, giving it a key role in the prevention of cancer, neurodegeneration, cardiomyopathy, diabetes, liver disease, and other diseases (75, 76). Excess NETs in ARDS are harmful, and elevated autophagy may reduce NETs to improve ARDS. However, progress in assessing the role of autophagy in human disease and its treatment relies heavily on the development of methods to monitor human autophagic activity (77).

Anti-PD-L1 antibodies are widely used in clinical practice for tumor treatment. Atezolizumab (a monoclonal antibody against PD-L1), which restores anticancer immunity, improved overall survival in patients with previously treated lung cancer or breast cancer and also showed clinical benefit when combined with chemotherapy as first-line treatment of lung cancer or breast cancer (78, 79). PD-L1 blockade can exert a protective effect on sepsis and the employment of anti-PD-L1 antibodies may be a promising therapeutic strategy for sepsis-induced immunosuppression (34, 35, 80). Clinical studies demonstrate that anti-PD-L1 peptide reverses T-cell dysfunction and improves survival in sepsis patients (81, 82). Notably, anti-PD-L1 antibodies, as immune checkpoint inhibitors, can reverse immunosuppression and may increase immune lung damage. It is then worth exploring whether the use of anti-PD-L1 antibodies increases the occurrence of septic lung injury (83–85). However, Thanabalasuriar et al. reported that blocking PD-L1 reduces airway inflammation and systemic treatment of injured animals with an anti-PD-L1 antibody prevented neutrophil accumulation in the lung and reduced susceptibility to infection (63). We verified the protective effect of anti-PD-L1 antibody against lung injury, the reduction of NET release and enhanced autophagy *in vivo* using a mice ARDS model. In addition, we confirmed that anti-PD-L1 antibody could produce the similar effects in reducing release of NETs and enhancing autophagy in human neutrophils from ARDS patients, which indicates the therapeutic effect of anti-PD-L1 antibody against ARDS and brings a new direction to the treatment of ARDS.

## Conclusion

In conclusion, PD-L1 has been identified as a therapeutic target against NET release and acute lung injury *via* regulating autophagy through PI3K/Akt/mTOR pathway in neutrophils. PD-L1 inhibition in neutrophils exerts a protective effect on ARDS by preventing excessive NETs. Therefore, anti-PD-L1 antibody administration may be a promising therapeutic strategy for sepsis-induced ARDS.

## Data availability statement

The original contributions presented in the study are included in the article/**Supplementary Materials**. Further inquiries can be directed to the corresponding authors.

## Ethics statement

The studies involving human participants were reviewed and approved by The study protocol was approved by the Committee on Ethics of Biomedicine Research in Naval Medical University, Shanghai, China. The patients/participants provided their written informed consent to participate in this study. The animal study was reviewed and approved by All animal studies were approved by the Committee on Ethics of Biomedicine Research in Naval Medical University, Shanghai, China. Written informed consent was obtained from the owners for the participation of their animals in this study.

## Author contributions

C-LZ and JX performed the research and analyzed the data. C-LZ and Z-ZZ wrote the major part of the manuscript. J-FW, X-MD, J-JB designed the research, ensured correct analysis of the data, and wrote the manuscript. All authors read approved the final manuscript. C-LZ, JX, Z-ZZ, PL, QL, YG, YM, X-JW, J-JB, X-MD and J-FW assisted in the design of the research, oversaw the collection of the data, and contributed to the writing of the manuscript. All authors critically revised the manuscript and gave final approval of the manuscript.

## Funding

This work was supported by the Natural Science Foundation of China (82072147, 81772105), Shanghai Rising-Star Program (21QA1411800), Top Project of the Youth Cultivation Program in PLA (20QNPY042).

## Conflict of interest

The authors declare that the research was conducted in the absence of any commercial or financial relationships that could be construed as a potential conflict of interest.

## Publisher’s note

All claims expressed in this article are solely those of the authors and do not necessarily represent those of their affiliated organizations, or those of the publisher, the editors and the reviewers. Any product that may be evaluated in this article, or claim that may be made by its manufacturer, is not guaranteed or endorsed by the publisher.
