# Corrigendum: PD-L1 maintains neutrophil extracellular traps release by inhibiting neutrophil autophagy in endotoxin-induced lung injury

**DOI:** 10.3389/fimmu.2022.1038083

**Published:** 2022-10-06

**Authors:** Cheng-long Zhu, Jian Xie, Zhen-zhen Zhao, Peng Li, Qiang Liu, Yu Guo, Yan Meng, Xiao-jian Wan, Jin-jun Bian, Xiao-ming Deng, Jia-feng Wang

**Affiliations:** Faculty of Anesthesiology, Changhai Hospital, the Naval Medical University, Shanghai, China

**Keywords:** ARDS, PD-L1, autophagy, neutrophils, neutrophil extracellular traps, anti-PD-L1 therapy

In the published article, there was an error in **Figure 6** as published. In **Figure 6B**, the integrated optical density ratio of p-p85/p85, p-Akt/Akt and p-mTOR/mTOR values do not correspond to the experiment group. The corrected **Figure 6** and its caption appear below.

**Figure 6:**
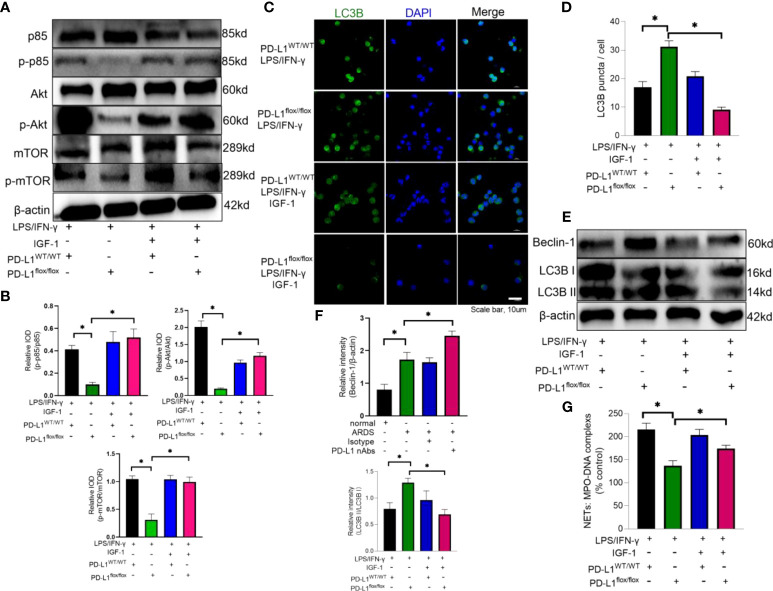
IGF-1 is able to counteract the protective effect of PD-L1 knockdown by activating the PI3K/Akt/mTOR pathway. **(A–G)** Neutrophils from PD-L1^WT/WT^ mice or PD-L1^flox/flox^ mice stimulated with IFN-γ (10ng/ml) and LPS (1μg/ml) are treated with IGF-1 (10ng/ml) or DMSO for 21h. **(A)** IGF-1 can activate the PI3K/Akt/mTOR pathway confirmed by Protein levels of p85, p-p85, Akt, p-Akt, mTOR, p-mTOR in neutrophils. **(B)** Integrated optical density ratio of p-p85/p85, p-Akt/Akt, P-mTOR/mTOR. **(C)** Autophagy induction assessed with LC3B staining (confocal microscopy; green: LC3B; blue: DNA) in neutrophils (scale bar: 10um). **(D)** LC3B puncta/cell are depicted. **(E)** PD-L1, Beclin-1 and LC3B II/I immunoblotting in neutrophils. **(F)** Integrated optical density ratio of LC3B II/LC3B I.**(G)** MPO-DNA complex measured in NETs structures in neutrophils culture supernatant. The values presented are mean ± SEM (n=6; *P<0.05, one-way analysis of variance).

The authors apologize for this error and state that this does not change the scientific conclusions of the article in any way. The original article has been updated.

## Publisher’s note

All claims expressed in this article are solely those of the authors and do not necessarily represent those of their affiliated organizations, or those of the publisher, the editors and the reviewers. Any product that may be evaluated in this article, or claim that may be made by its manufacturer, is not guaranteed or endorsed by the publisher.

