# Supplementary material for: PD-L1 maintains neutrophil extracellular traps release by inhibiting neutrophil autophagy in endotoxin-induced lung injury
Source: Front Immunol. 2022 Aug 9;13:949217. doi: 10.3389/fimmu.2022.949217 (PMC9396256; doi:10.3389/fimmu.2022.949217)
Supplement: Supplementary file 1 [file DataSheet_1.docx]

**Supplemental Figure Legends**

**Figure** **S1**. **Genetic deletion of PD-L1 in mice neutrophils can reduce NETs release**. (A-B) Neutrophils from PD-L1^WT/WT^ mice or PD-L1^flox/flox^ mice are stimulated with IFN-γ (10ng/ml) and LPS (1μg/ml) for 21 hours. (A) Representative immunofluorescence images of Cit H3 (green) and MPO (red) staining with blue DAPI nuclear staining in mice neutrophils. Neutrophils express MPO (red) and NETs forming neutrophils also express Cit-H3 (green). Cyan fluorescence represents the colocalization of Cit-H3 with DNA. The red arrows to neutrophils making NETs. The scale bar indicates 50 μm. Higher magnification images are shown lower row of figures – scale bars indicate 10 μm. (B) MPO-DNA complex measured in NETs structures in neutrophils culture supernatant. The values presented are mean ± SEM (n=3; *P<0.05, one-way analysis of variance).

**Figure S2. Inhibition of autophagy attenuates the effect of knockdown of PD-L1 to reduce the release of NETs *in vivo*.** (A-B) ARDS mice were injected intraperitoneally with wortmannin (1.5mg/kg). (A) Representative immunofluorescence images of Cit-H3 (green) and MPO (red) staining with blue DAPI nuclear staining in lungs. Neutrophils express MPO (red) and NETs forming neutrophils also express Cit-H3 (green). Cyan fluorescence represents the colocalization of Cit-H3 with DNA. The white arrowheads point to neutrophils not making NETs and the red arrows to neutrophils making NETs. The scale bar indicates 20 μm. Higher magnification images are shown lower row of figures – scale bars indicate 10 μm. (B) MPO-DNA complex measured in NETs structures in BALF. (n=3; *P<0.05, one-way analysis of variance).

**Figure S3. Inhibition of autophagy attenuates the effect of knockdown of PD-L1 to reduce the release of NETs *in vitro*.** (A-B) Neutrophils from PD-L1^WT/WT^ mice or PD-L1^flox/flox^ mice stimulated with IFN-γ (10ng/ml) and LPS (1μg/ml) are treated with Wortmannin (1uM) or DMSO for 21hours. (A) Representative immunofluorescence images of Cit H3 (green) and MPO (red) staining with blue DAPI nuclear staining in neutrophils. Neutrophils express MPO (red) and NETs forming neutrophils also express Cit-H3 (green). Cyan fluorescence represents the colocalization of Cit-H3 with DNA. The red arrows to neutrophils making NETs. The scale bar indicates 50 μm. Higher magnification images are shown lower row of figures – scale bars indicate 10 μm. (B) MPO-DNA complex measured in NETs structures in neutrophils culture supernatant. The values presented are mean ± SEM (n=6; *P<0.05, one-way analysis of variance).

**Figure S4. Anti-PD-L1 antibody can increase autophagy levels in human neutrophils.** Neutrophils from ARDS patients were treated with anti-PD-L1 antibody (10ug/10^6^ cells) for 24h. (A) Autophagy induction assessed with LC3B staining (confocal microscopy; green: LC3B; blue: DNA) in neutrophils (scale bar: 10um). (B) LC3B puncta/cell are depicted. The values presented are mean ± SEM (n=3; *P<0.05, one-way analysis of variance).

**Supplemental Figures**

**Figure S1**

**
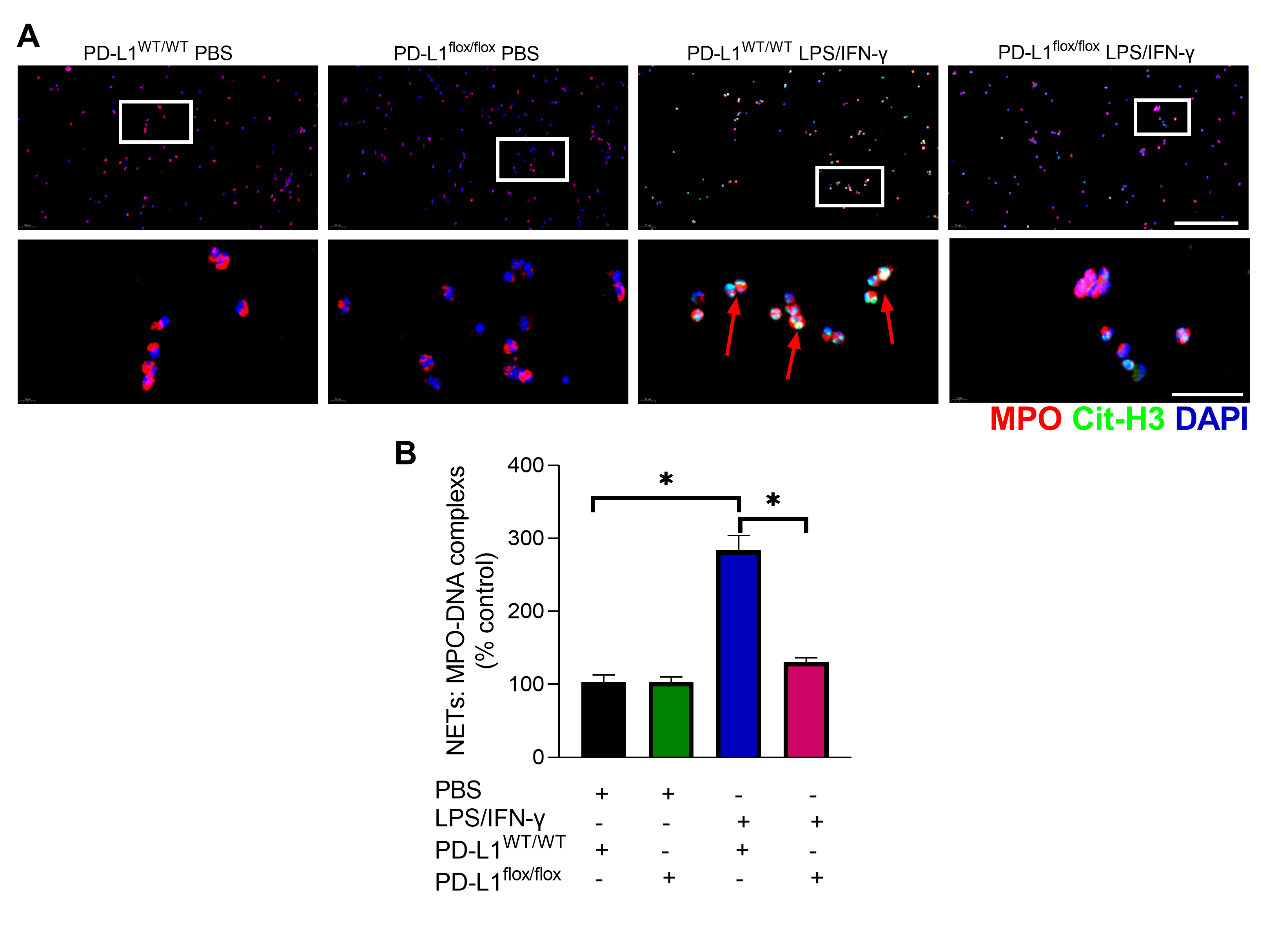
**

**Figure S2**


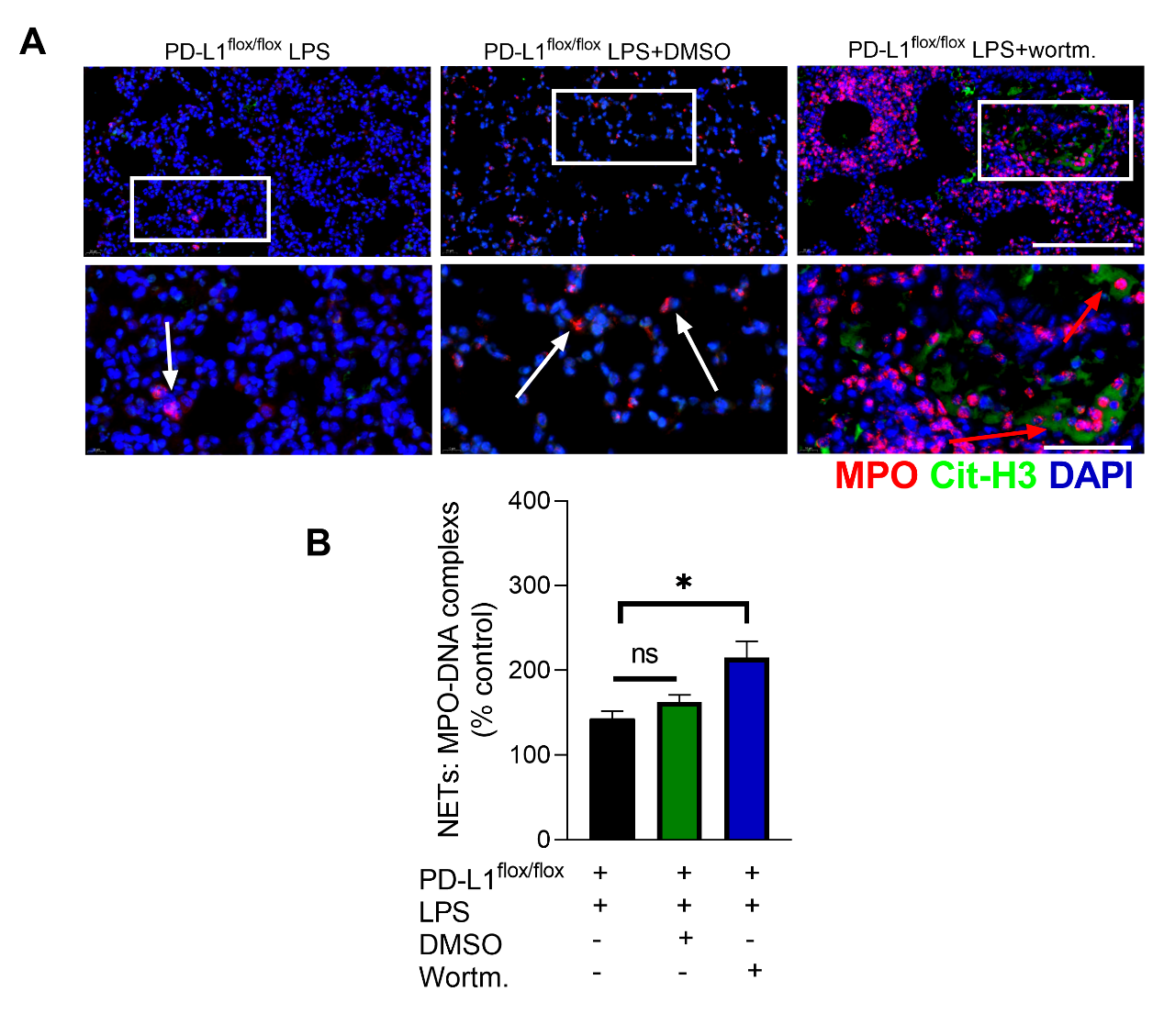


**Figure S3**

**
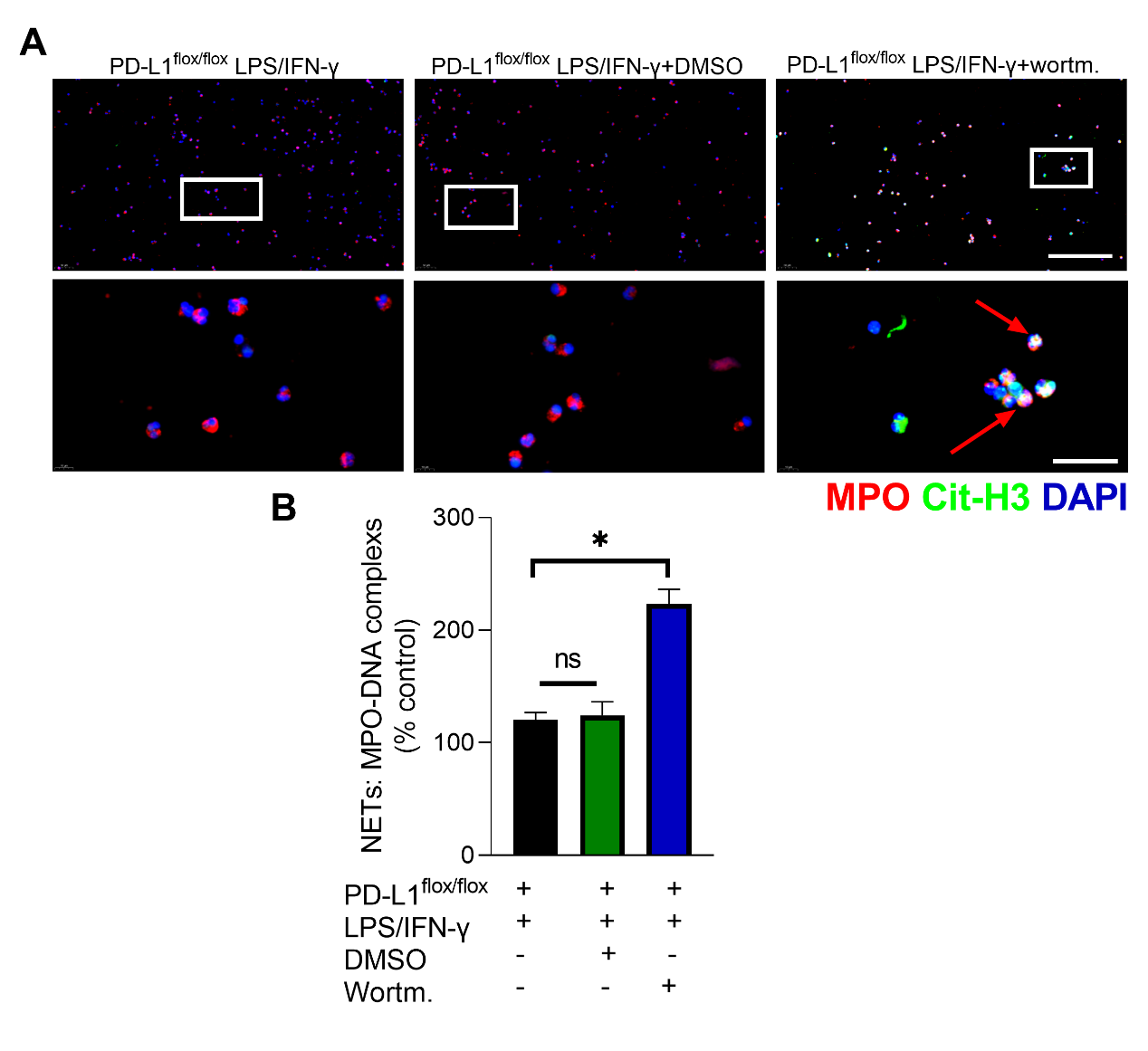
**

**Figure S4**

**
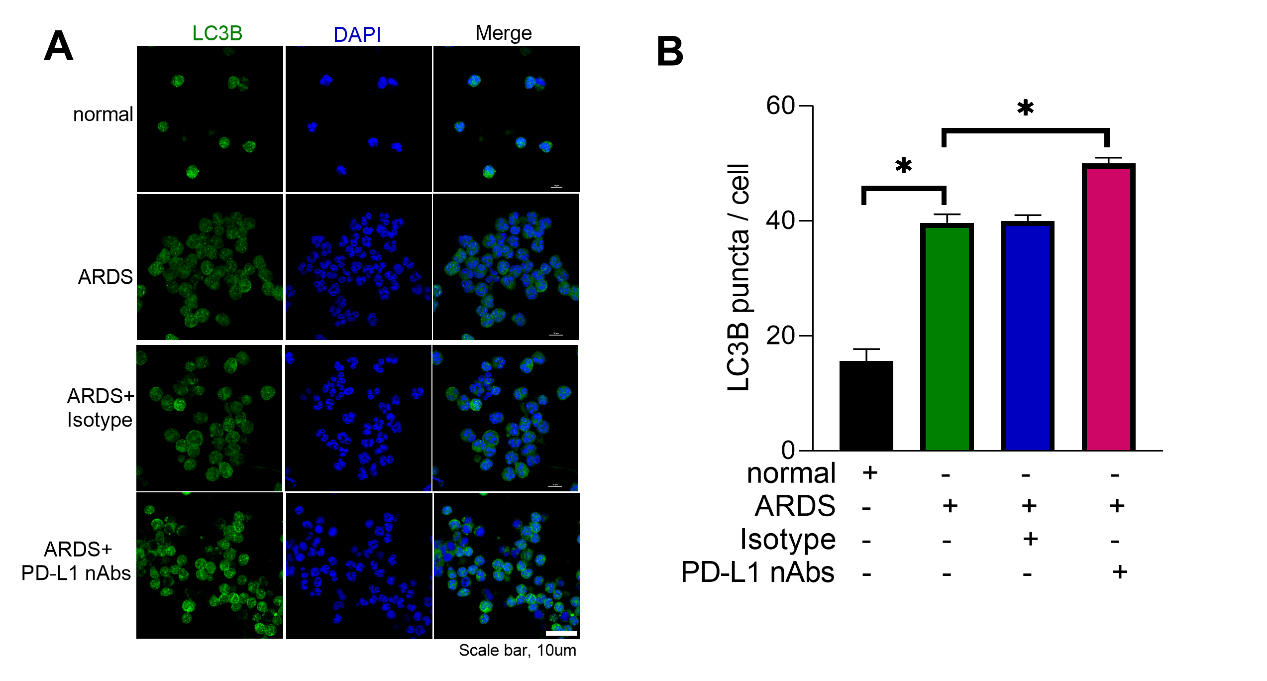
**
